# Determination of protoplast growth properties using quantitative single-cell tracking analysis

**DOI:** 10.1186/s13007-022-00895-x

**Published:** 2022-05-18

**Authors:** Jonathan Dawson, Saurabh Pandey, Qiuju Yu, Patrick Schaub, Florian Wüst, Amir Bahram Moradi, Oleksandr Dovzhenko, Klaus Palme, Ralf Welsch

**Affiliations:** 1 Institute of Biology II, Faculty of Biology, Albert-Ludwigs-University of Freiburg, Schänzlestrasse 1, 79104 Freiburg, Germany; 2 Institute of General Electrical Engineering, University of Rostock, Albert-Einstein-Str. 2, 18059 Rostock, Germany; 3 ScreenSYS GmbH, Engesserstr. 4, 79108 Freiburg, Germany; 4 BIOSS Center for Biological Signaling Studies, Albert-Ludwigs-University of Freiburg, Schänzlestrasse 1, 79104 Freiburg, Germany; 5 State Key Laboratory of Crop Biology, College of Life Sciences, Shandong Agricultural University, Daizong Street 61, Tai’an, 271018 China; 6 Present Address: Augusta University, 1201 Goss Ln, Augusta, GA 30912 USA

**Keywords:** BAG, Cell expansion, Cell tracking, Protoplasts, Single cell, Tobacco, Tracking

## Abstract

**Background:**

Although quantitative single-cell analysis is frequently applied in animal systems, e.g. to identify novel drugs, similar applications on plant single cells are largely missing. We have exploited the applicability of high-throughput microscopic image analysis on plant single cells using tobacco leaf protoplasts, cell-wall free single cells isolated by lytic digestion. Protoplasts regenerate their cell wall within several days after isolation and have the potential to expand and proliferate, generating microcalli and finally whole plants after the application of suitable regeneration conditions.

**Results:**

High-throughput automated microscopy coupled with the development of image processing pipelines allowed to quantify various developmental properties of thousands of protoplasts during the initial days following cultivation by immobilization in multi-well-plates. The focus on early protoplast responses allowed to study cell expansion prior to the initiation of proliferation and without the effects of shape-compromising cell walls. We compared growth parameters of wild-type tobacco cells with cells expressing the antiapoptotic protein Bcl2-associated athanogene 4 from Arabidopsis (*AtBAG4).*

**Conclusions:**

*AtBAG4*-expressing protoplasts showed a higher proportion of cells responding with positive area increases than the wild type and showed increased growth rates as well as increased proliferation rates upon continued cultivation. These features are associated with reported observations on a BAG4-mediated increased resilience to various stress responses and improved cellular survival rates following transformation approaches. Moreover, our single-cell expansion results suggest a BAG4-mediated, cell-independent increase of potassium channel abundance which was hitherto reported for guard cells only. The possibility to explain plant phenotypes with single-cell properties, extracted with the single-cell processing and analysis pipeline developed, allows to envision novel biotechnological screening strategies able to determine improved plant properties via single-cell analysis.

**Supplementary Information:**

The online version contains supplementary material available at 10.1186/s13007-022-00895-x.

## Background

Plant cells are organized within a tissue context in which most cells are embedded in a stiffened chassis, the cell wall, comprising cellulose microfibrils which interact with a matrix of mainly pectins, hemicelluloses and structural proteins [43]. This chassis is flexible during early plant cell developmental stages which take place in restricted regions within a plant body, the meristems. These zones contain stem cells with a high proliferation activity which generate small, isodiametric, undifferentiated cells, subsequently increasing in size by about tenfold, to finally differentiate into specialized plant cells, for instance mesophyll cells of leaves, pavement cells of the epidermis, or endodermis cells of roots [2, 43].

Usually, proliferation and subsequent cell volume increase is restricted to the early stages of cell development prior to the stiffening of cell wall components. However, plant cells are considered as totipotent, which is defined as the capacity of differentiated cells to first dedifferentiate, then proliferate and redifferentiate into the complete repertoire of plant cell identities thus forming whole plants [22]. This process, however, requires suitable stimuli which can occur naturally, e.g. induced by wounding of plant tissues which finally results in the formation of new organs [17]. Dedifferentiation can also be induced in isolated, differentiated single cells, but requires presence of suitable media components and plant growth regulators and is stimulated by stochastic gene expression induced through the isolation process [14, 53, 58]. For isolation, cell walls are usually lysed enzymatically and single cells, protoplasts, are released. Protoplasts are extensively used in plant research for multiple purposes, recently extending towards gene editing approaches and single-cell transcriptomics and genomics [48, 50].

Besides biotechnological applications, this system offers the advantage of studying growth properties independent from the cell wall counterforce as cell walls are regenerated earliest after about 48 h [35]. Upon isolation and the provision of suitable physiological conditions in the medium, a proportion of protoplasts remains viable and can grow in size [23, 55]. Cell growth can be influenced by multiple factors, both external, such as chemical and physical stimuli, as well as internal, such as the cell size and physiology at the time of cell extraction and isolation from in vivo to in vitro environment [1, 12]. Even within a population of isogenic cells there is a high variability in growth rates of individual cells [49, 55]. Cell growth analysis is frequently done at the cell population level at a single time point, and is thus an end-point analysis. Such population-based studies are informative for effects of chemicals or other factors on average cell growth, but hide information on the effects on individual cells and their developmental properties occurring over time. Thus, average growth responses at the population level may not translate to responses at the level of single cells. In addition, single time point analysis cannot relate the differences in growth responses of single cells to their initial state [12].

Single-cell tracking allows to study changes in single-cell properties, such as its size, shape and relate with intracellular metabolic state as well as gene expression over time [51]. This provides a quantitative approach to study the influence of various perturbations on single-cell dynamics, such as cell growth and proliferation rate, and to understand how these processes are regulated. Single-cell tracking complemented with high-content image-based data from high-throughput microscopes is very powerful in providing deep insight into cellular dynamics through large chemical screening, statistically rigorous correlation studies and modeling [33, 39]. However, analysis of such high-volume data in a time-efficient manner necessitates the development of computational tools capable of efficient processing of images and subsequent statistical data analysis which we approached in this work.

Our approach is based on the fact that quantitative microscopic analysis of single-cell responses is well established and widely used for animal and yeast cells while corresponding applications on plant single cells is largely restricted only to the very early time points occurring within minutes to a few hours after isolation [6, 19, 33, 52]. However, monitoring of growth properties over time, several days after isolation until the initiation of proliferation, has only been rarely done so far [35]. This is probably due to difficulties associated with the tracking of isolated protoplasts which tend to move and aggregate when cultivated in liquid media. Moreover, quantification of cell area changes over time requires the precise determination of cell shapes from bright field image acquisitions. However, the tools to convert a greyscale image into a corresponding binary image established for animal and yeast cells are unsuited to be applied on plant cells with their different morphology.

In this study we have used a method of immobilizing protoplasts in high density in multi-well plates coupled with repeated image acquisitions using automated microscopy in order to quantify area changes of single cells during continued incubation. We present the development of an image processing pipeline including image processing requirements needed to precisely quantify area changes of single cells. Our processing routine was applied on isolated protoplasts from tobacco leaves as one of the most widely used systems [37]. Moreover, we challenged our system by including protoplasts from tobacco plants expressing the antiapoptotic protein BCL2-ASSOCIATED ATHANOGENE 4 (*BAG4)* from Arabidopsis which was reported to affect various plant processes ranging from altered stress response to increased stomatal movements [29]. Among different plant cell identities, stomatal guard cells have a special requirement for an efficient regulation of ion homeostasis as they need to quickly adjust their shapes to fulfil their function as a valve regulating water and gas exchange in response to changing physiological conditions. Potassium influx in stomatal guard cells is mainly determined by two K^+^ channel family members, KAT1 and KAT2 [26, 38]. Accordingly, the KAT1 channel is subjected to a large number of regulatory mechanisms, e.g. via phosphorylation or its transport activity [45]. Recently, Locascio et al. [29] identified BAG4 as another interaction partner of KAT1. They showed that BAG4 controls the abundance of KAT1 on plasma membranes of stomatal guard cells, which affects stomatal movements, however, it remained unknown whether BAG4 also affects the physiology of other cells. We questioned whether a similarly altered ion homeostasis might affect expansion properties of isolated protoplasts of *BAG4-*expressing plants and applied an automated process for quantitative analysis. Comparative determination of the properties of wild-type versus *BAG4-*expressing tobacco cells revealed a universal function of *BAG4* in regulating cellular volume changes which seems not restricted to guard cells. Moreover, our approach is suitable to be applied to precisely determine single-cell area changes in plant protoplasts and allows to align single-cell responses with phenotypic alterations at the whole plant level. This opens the way to envision an improvement of plant properties by single-cell screening procedures as novel high-throughput breeding strategy.

## Results

### Establishment of protoplast tracking assay

For the establishment of a system which allows to determine single cell properties for unraveling possible effects of BAG4 on cell expansion properties, we overexpressed *BAG4* in tobacco. We generated independent transgenic lines expressing Arabidopsis *BAG4* (*AtBAG4*) and AtBAG4 as N-terminal translational fusion with YFP (YFP-BAG4), respectively. The expression of the YFP-BAG4 fusion protein allowed us to monitor AtBAG4 localization properties simultaneously. The transgene expression levels were determined by qRT-PCR (see Additional file 2: Fig. S1) and the two independent strongest *AtBAG4*-expressing lines (lines #1-1-1 and #2-20-5) were chosen for further analysis.

**Fig. 1:**
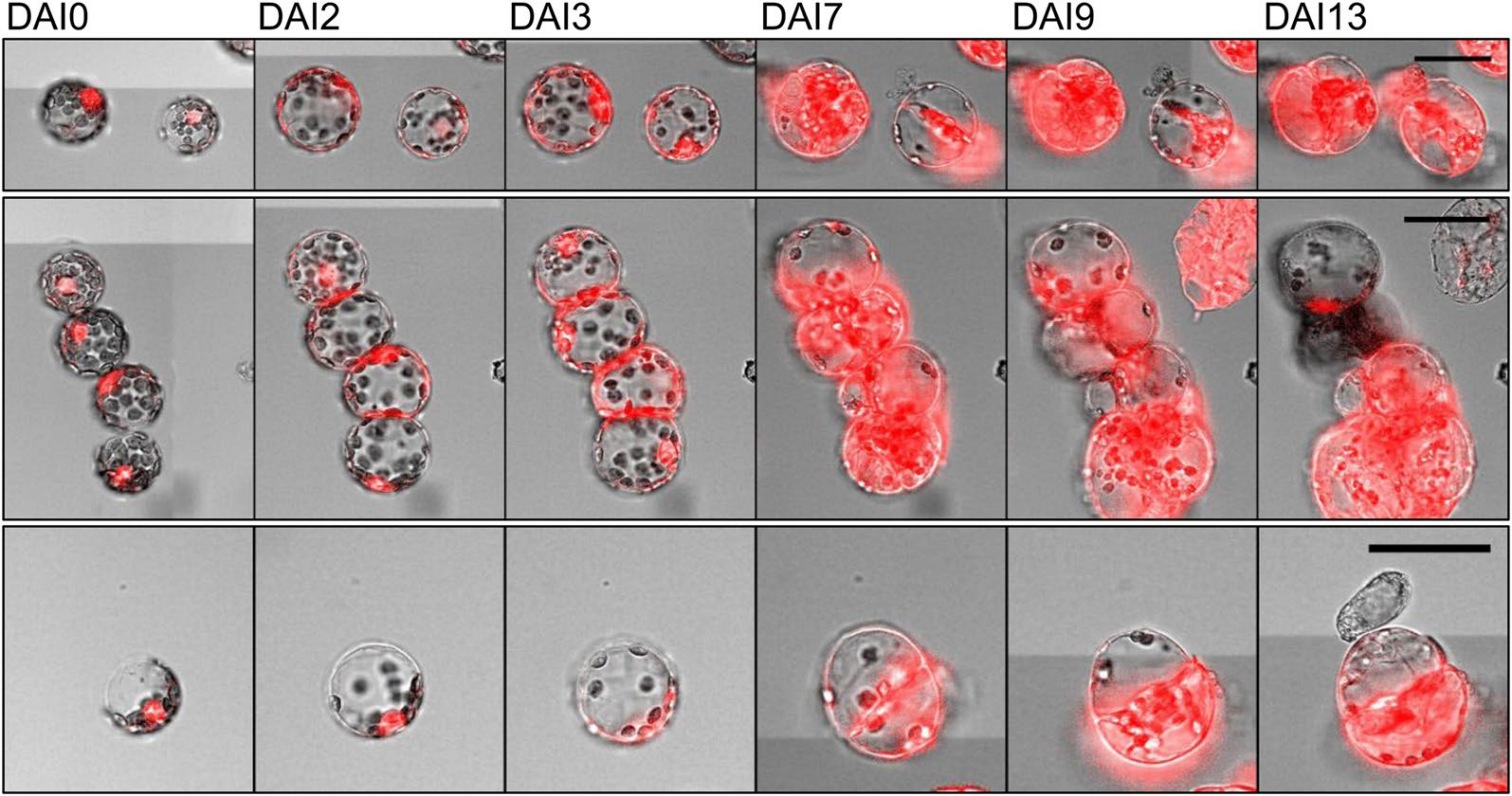
AtBAG4 localization during proliferation. *Nicotiana tobacum* protoplasts expressing *YFP-AtBAG4* were immobilized and recorded in bright field and epifluorescence modes for 13 days after immobilization (DAI). While YFP-AtBAG4 is almost exclusively nuclear localized immediately after isolation at DAI0, YFP-AtBAG4 is found also in cytoplasmic membranes with continued incubation until DAI3 and increases massively in microcalli after completion of cell divisions. Note also the increased fluorescence in contact areas of adjacent protoplasts. Bar = 50 µm

By monitoring growth properties of protoplasts over time, we explored to establish a microscopy workflow which allowed cell tracking analysis. For this, we isolated protoplasts from young leaves of in vitro cultivated tobacco plants. This yielded almost quantitatively mesophyll protoplasts with only minor abundance of other leaf cell types such as guard cells or epidermal cells. After isolation, protoplasts were immobilized into multi-well-plates with a density of 8000 protoplasts per well. Immobilization was achieved by the use of low-melting agarose which allowed to position most cells in a monolayer at the bottom of the wells by low-speed centrifugation. Subsequently the agarose block was overlaid with liquid proliferation medium and cell layers were repeatedly recorded over time using automated microscopy. The proliferation medium contained the plant growth regulators auxin and cytokinin required to induce expansion and proliferation of protoplasts [47]. We first exploited YFP as a fluorescent marker to monitor AtBAG4 localization, and recorded single-cell development during 13 days after immobilization (DAI, Fig. 1). Interestingly, YFP-AtBAG4 was almost exclusively localized in the nucleus/ER immediately after protoplast isolation/immobilization, while with continued cultivation, the YFP-AtBAG4 signal was present in cytoplasmic membranes in addition. This agrees with the observation that the KAT1-AtBAG4 complex colocalized with an ER marker which was associated with the function of AtBAG4 in KAT1 assembly prior to its transport to plasma membranes [29]. Moreover, YFP-AtBAG4 signal intensity increased also in areas of contacting protoplasts. Protoplast area increased visibly during the first 3 DAIs without visible cell divisions, while after 7 DAIs, protoplasts initiated cell proliferation. Strikingly, with the beginning of cell divisions, YFP-AtBAG4 signal intensity increased strongly and accumulated preferably in the cell plate in early cell divisions, while it was detected throughout the cytoplasm with the completion of divisions and subsequent proliferations at early microcalli stage (Fig. 1).

### Development of single cell tracking workflow

**Fig. 2:**
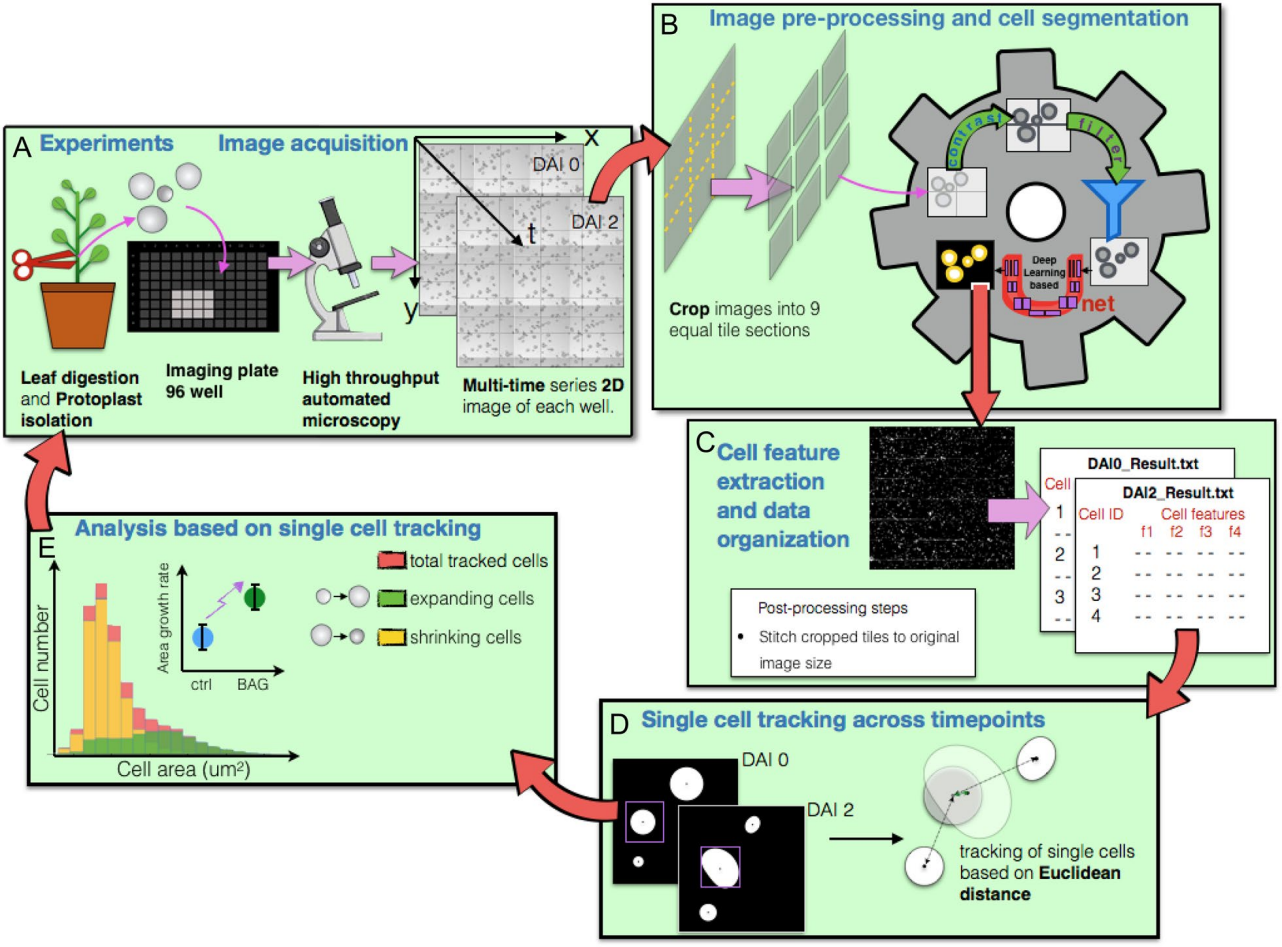
General workflow of automated image processing and data analysis pipeline. **A** Isolated protoplasts are immobilized into 96-well plates and subjected to automated microscopy with daily recordings in bright field mode for 4 days after immobilization (DAIs). Chronological image stacks are generated and subjected to positional correction of slight cell shifts occurring during repeated recordings. **B** Bright field images are cropped into 9 equal tile sections in order to accelerate further image processing. After preprocessing cell segmentation is done by U-net. This generates result files listing all the identified cells and their corresponding features such as cell position coordinates, area and circularity (**C**). **D** After removal of cell clusters and contacting cells, the result file of each well at each time point is used to track individual single cells using Euclidean distance of cell centroid between two time points. **E** Using this result file, statistical data analysis is carried out to build cell area distribution of cells tracked between two time points. The results of rigorous statistical analysis is used to infer the effect of different conditions on cell behavior, such as expansion and proliferation, and guide future experiments

These results showed that the immobilization method used was suitable to maintain viable tobacco protoplasts after immobilization and allowed protoplast proliferation. In order to quantify area changes of single cells tracked over time, an automatized image processing and data analysis pipeline was developed (Fig. 2). We assumed that turgor-mediated single-cell area changes are slowed down as soon as cell proliferation is initiated, as this is accompanied by the generation of cell plates and the formation of shaping cell walls thus forming multicellular microcalli (Fig. 1). Therefore, in order to focus on single-cell area changes over time, we restricted the analysis on the initial three days following protoplast isolation, which was prior to first cell divisions. After immobilization, protoplasts were recorded daily in each well in bright field mode and cell positions were aligned among all chronological images in order to correct for slight positional displacements of protoplasts using the template matching and slice alignment plugin with ImageJ [56]. Next, in order to detect single cells and precisely measure their shapes, we segmented all images using U-net, a deep-learning-based software package for cell detection and segmentation [10].

**Fig. 3:**
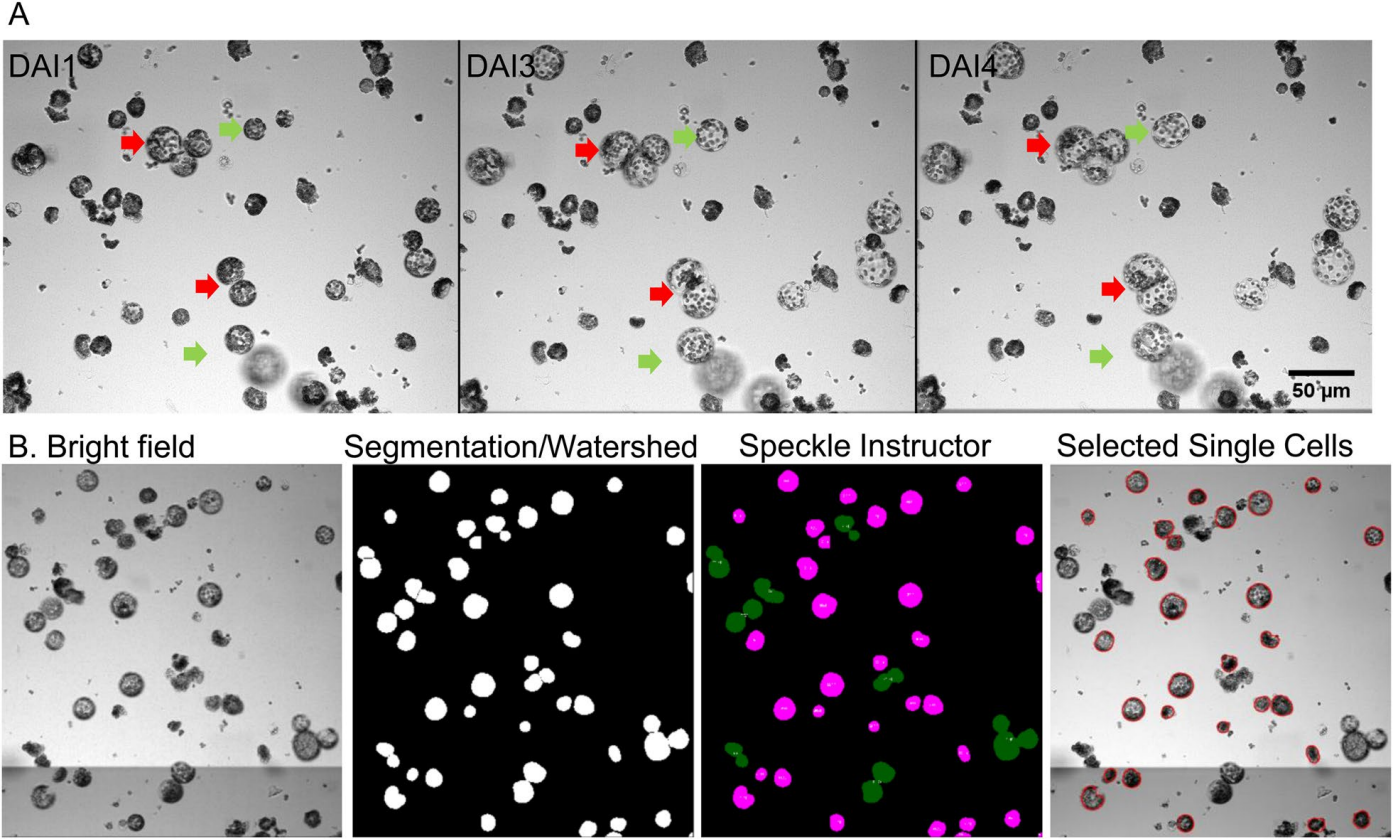
Selection of isolated single cells for tracking analysis. Bright field images of a time series of immobilized tobacco wild-type protoplasts, recorded at 1, 3 and 4 days after immobilization (DAI). **A** Protoplast growth is affected by contact with neighboring cells if positioned too close to each other (red arrow) while only single cells are able to expand isodiametrically (green arrow). **B** The property of segmented cell clusters to split into multiple objects after application of the watershed algorithm was exploited by adapting the Speckle Instructor plugin to filter for single cells exclusively

Depending on their proximity, expanding single cells frequently contact each other and form a cluster which poses difficulties in addressing their single-cell identity and alters their growth properties similar to proliferating protoplasts mentioned above (Fig. 1). We therefore intended to exclude contacting protoplasts from the analysis. In order to automatize exclusion of clustering cells, we exploited the speckle inspector plugin in ImageJ which is initially intended to quantify smaller features/speckles within larger objects, i.e. number of nuclei within cells [5]. For our purpose, we first sliced cell clusters formed by contacting cells into multiple fragments applying the watershed algorithm on segmented images (Fig. 3). Then, we adopted the speckle inspector plugin in ImageJ to compare the original segmented image with the corresponding watershed image in order to identify cells which formed clusters [4]. This allowed removing these cell clusters from further analysis leaving only individual single cells that were tracked between time points.

**Fig. 4:**
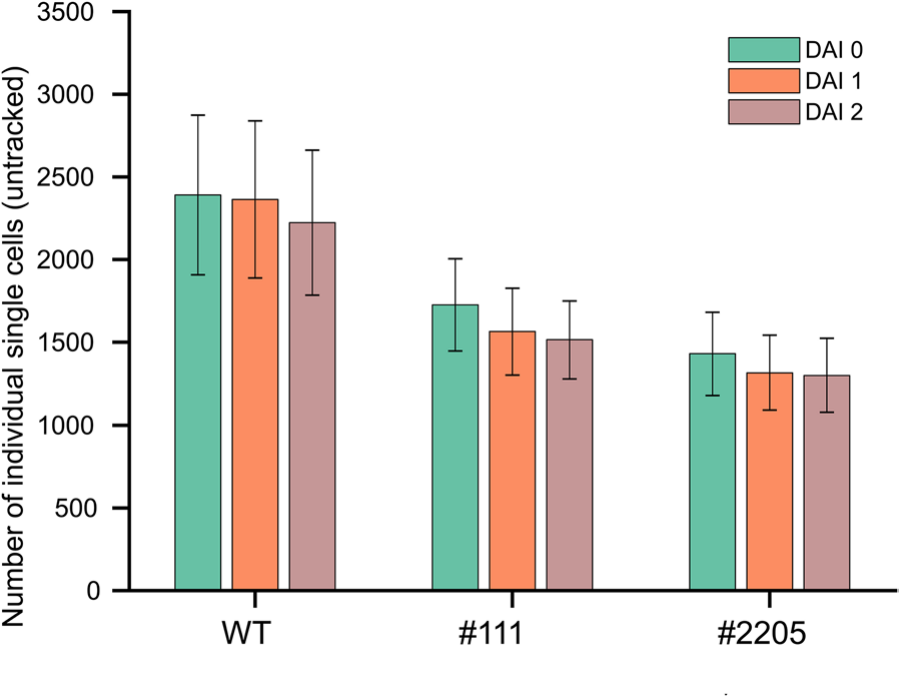
Total number of cells passing cluster filtration. Immobilized tobacco protoplasts were subjected to filtration step removing clustered cells which appeared over time due to contacting protoplasts after volume increase. Cells numbers are shown for wild-type (WT) and the two *AtBAG4*-expressing lines #1-1-1 and #2-20-5 recorded at DAI0, DAI1 and DAI2. The total number of cells drops slightly over time indicating cluster formation. Data are mean ± SD number of cells per well for 3 bioreps with 3 tech reps each

From a total number of 8000 protoplasts per well, about 2500 cells finally passed all filtration steps and were used for further quantitative cell expansion analysis. As shown in Fig. 4, the average individual cell numbers passing the filtration decreased slightly with continued incubation, explained by the elimination of cells forming clusters caused by volume increases over time. This drop in individual cell numbers occurred at DAI2 for wild-type protoplasts and already after DAI1 for *AtBAG4-*expressing lines, but then remained constant.

### Single-cell population analysis

In order to track cell lineages of individual cells over time and also to consider possible slight positional cell shifts occurring between image acquisitions of subsequent time points, we tracked segmented cells retrogradely based on Euclidean distance. Identification of identical cells in each images in a time series is realized by calculating the distance between the centroids of cell pairs. A threshold of 27 µm shift was allowed for a cell being tracked across two time points. All further analyses were performed with data extracted from these tracked cell lineages.

**Fig. 5:**
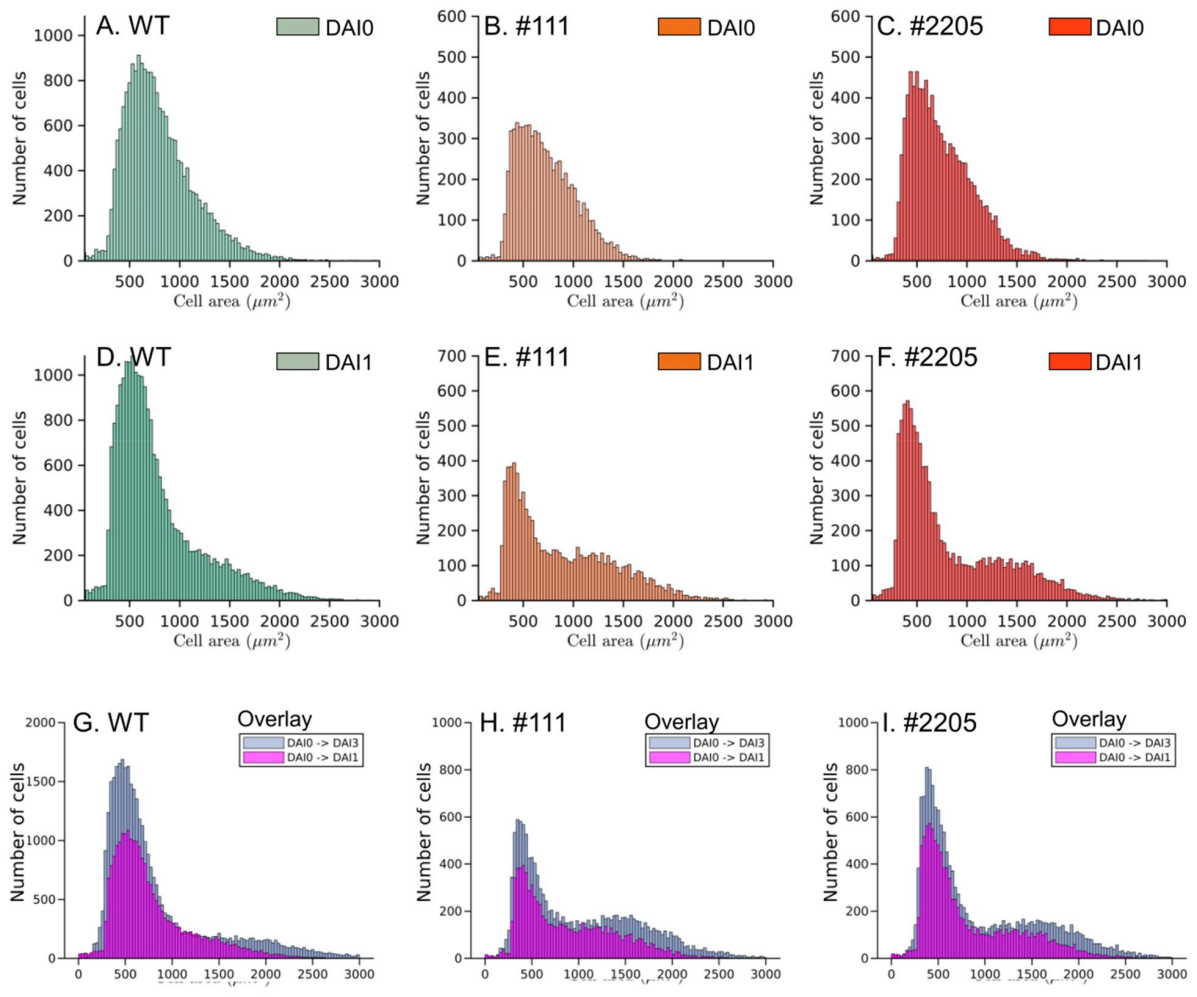
Area distribution of isolated protoplasts. Tobacco protoplasts were immobilized, filtered for individual single cells, tracked across DAI0 and DAI1, and subjected to size distribution analysis. Cell area distribution of individual single cells at DAI0 (**A**–**C**) and DAI1 (**D**–**F**) for **A** wild-type and *AtBAG4*-expressing lines #111 (**B**) and #2205 (**C**). An overlay of the area distribution of cells tracked between DAI0 and DAI1 (purple) and cells tracked between DAI0 and DAI3 (light green) is shown in **G**–**I**. Data shown are pooled data from 3 bioreps with 3 tech reps each

Growth of individual single cells was quantified by constructing the cell area distribution of tracked cells. The cells were tracked up to three DAIs in wild type and the two *AtBAG4*-expressing lines, namely lines #111 and # 2205. The cell area distribution of wild-type and *AtBAG4*-expressing cells at the initial starting time point, i.e., DAI0, formed a single, slightly asymmetric peak indicating higher area variability in larger cell areas (Fig. 5A–C). The average area of protoplasts was 850 $\pm$ 20 µm^2^ for wild type, 750 $\pm$ 10 µm^2^ for line #111 and 800 $\pm$ 40 µm^2^ for line #2205 which corresponds to average cell diameters of 16.45 $\pm$ 2.5 µm for wild type, 15.45 $\pm$ 1.8 µm for #111 and 16 $\pm$ 3.6 µm for line #2205 which matches with the average size of mesophyll protoplasts [15].

A plot of the cell area distribution of individual single cells at DAI1 revealed an additional second peak corresponding to a cell population which responded with increased cell areas (Fig. 5D–F). As expected, the peak of the DAI0 area distribution was reduced in width, however the abundance of cells with smaller cell areas was increased. This corresponds to the population of cells which responded with cell area decreases, thus shrinkage during the first day of incubation. Remarkably, the second peak appearing on DAI1 was more pronounced in the cells from *AtBAG4*-expressing lines compared to the wild type. This tendency continued also for the subsequent days upon immobilization, as visualized in Fig. 5G–I. The overlay of cell area abundances of individual tracked cells at DAI1 and DAI3 revealed a further shift of the cell population with increased cell areas while the peak corresponding to cells with shrinking cell areas further increased slightly in size.

### Single-cell response analysis

**Fig. 6:**
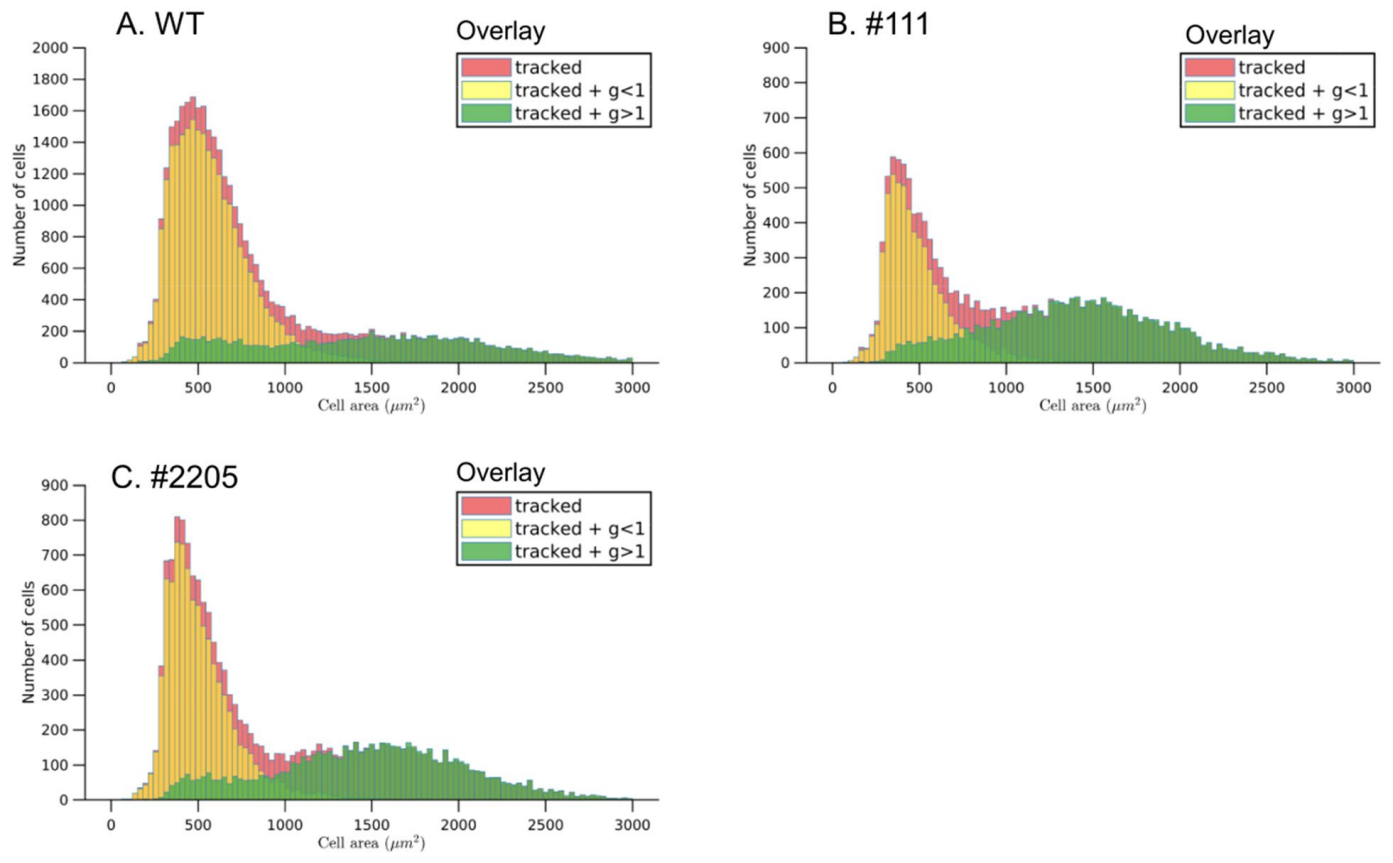
Area distribution and cell response of tracked single cells. The total cell area distribution (red) of single cells tracked between DAI0 and DAI1 in **a** wild-type, and *AtBAG4*-expressing lines **b** #111 and **c** #2205. The overlay indicates the area distribution of tracked cells whose growth rate is greater than 1 (green) or less than 1 (yellow). Data shown are pooled data from 3 bioreps with 3 tech reps each

In order to separate the two distinct cell populations responding with either increased or decreased cell areas, we used the information provided from the tracking analysis to determine cell area changes over time. Using growth rate, defined as the ratio of cell area at a later time point to cell area at DAI0, i.e., the first time point, we distinguished cells that had an area growth greater than 1 from cells which had an area growth lower than 1 (Fig. 6). An overlay of area distributions of the cells with growth rate greater 1 and less than 1 on the total cell area distribution confirmed that the total area distribution is composed of the two cell subpopulations with the two different responses defined above. The second peak in the total cell area distribution, located at the larger cell area values, is mainly due to cells with positive area growth, whereas, the first peak, located at smaller cell area values, is shaped by shrinking cells.

**Fig. 7:**
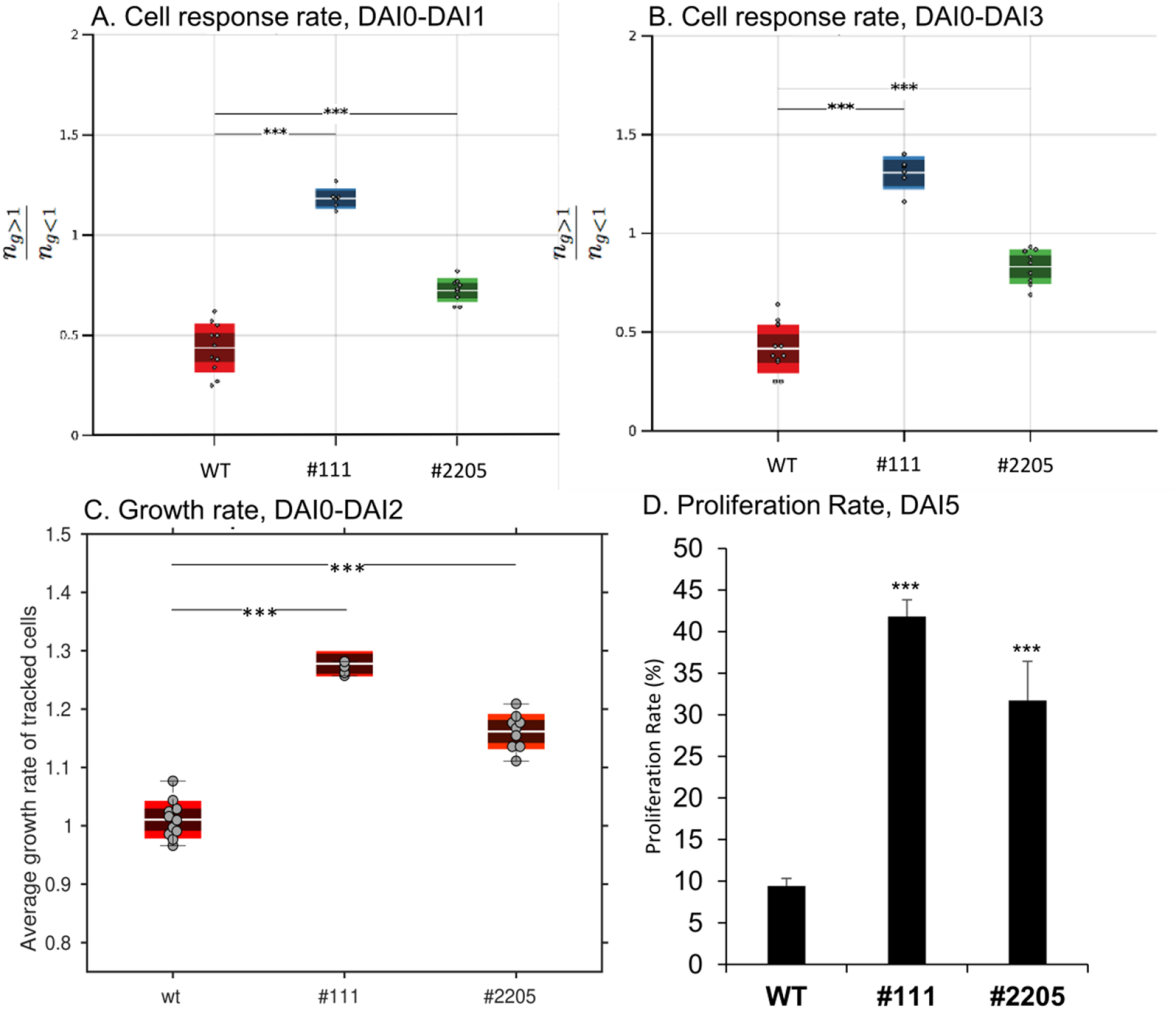
Quantification of cell responses and growth rates. The response rate *n* defines the ratio of the number of cells with area growth rate greater than 1 to the number of cells with area growth rate less than 1. This was generated from individual single cells tracked between DAI0 and DAI1 (**A**) and DAI0 and DAI3 (**B**). Higher *n* values for *AtBAG4*-expressing protoplasts in comparison to wild type indicates a significantly increased pool size of expanding cells. **C** The cell growth rate was determined by quantification of the relative area changes between two subsequent time points which revealed about 20–30% larger growth rates for *AtBAG4*-expressing lines. The scattered points on each represents the mean of each biorep and tech rep separately. The white line in the middle of each box represents the overall mean (one way ANOVA, *** indicates p < 0.001). **D** Protoplast proliferation rate determined at DAI5 (3 bioreps ± SEM, ttest, *** indicates p < 0.001)

The differences in the shapes of the cell area distribution over time for wild-type and *AtBAG4*-expressing lines indicated different responses. The total cell area distributions show that the number of cells with smaller cell area values is lower in *AtBAG4*-expressing lines as compared to wild-type, whereas the number of cells with higher cell area values increases in *AtBAG4*-expressing lines. In order to quantify the different proportions of cells responding with negative and positive cell area changes over time, we computed for all cells tracked between two time points the quantity $n=\frac{{n}_{>1}}{{n}_{<1}}$ which is the ratio of the total number of cells with an area growth greater than 1 and the total number of cells with an area growth rate less than 1 (Fig. 7A, B). For wild-type protoplasts, *n* was about 0.5 and remained constant when quantified for cells tracked between DAI0 and DAI1 and between DAI0 and DAI3. This indicates that in the wild-type the cell population size responding with cell shrinkage upon isolation was twice as large as the cell population size responding with growth. In contrast, *AtBAG4*-expressing lines had higher *n* values of 0.8 (#2205) and 1.3 (#111), respectively which also remained constant during continued incubation. Thus, expression of *AtBAG4* strongly shifted the cell population size towards cells responding with growth compared to wild-type protoplasts. The constant ratios of *n* observed over time for all genotypes indicates that the decision for cell area increase or shrinkage is taken upon isolation and remains largely unchanged until DAI3.

These findings are visualized by a density scatter plot which showed, qualitatively, the influence of *AtBAG4* expression on the cell area growth in relation to their initial/starting size. Compared to wild-type protoplasts in both *AtBAG4*-expressing lines the cloud of data scatter spread to higher values of relative area growth, corroborating that higher number of cells respond with an area increase more than two-fold between DAI0 and DAI2 (Additional file 2: Fig. S2).

### Single-cell growth rates

The previous analysis allowed us to conclude on different growth properties of *AtBAG4-*expressing lines when individual single cells were monitored for three DAIs. While the population analysis suggested that a larger population of protoplasts responded with increased area changes than in wild type, the response analysis confirmed that more cells are viable and show positive area changes. However, both analysis did not clearly allow to conclude on the actual rate with which cell area change. This rate was determined by calculating the relative cell area change between two subsequently recorded time points of all tracked cell lineages (Fig. 7C). This revealed that wild-type protoplasts expanded with less than 5% area increase in average between two time points. In contrast, both *AtBAG4*-expressing lines showed between with 15–28% area change corresponding to 1.1 and 1.3-fold higher growth rates compared with the wild type.

Overall, our developed image analysis pipeline allowed to determine that protoplasts expressing *AtBAG4* have an increased response rate as well as an increased growth rate when monitored within the first three days following isolation. In order to investigate how this affects protoplast proliferation, we estimated the number of microcalli which revealed that *AtBAG4* expression increased the proliferation rate by 3–fourfold compared with wild-type protoplasts (Fig. 7D; Additional file 2: Fig. S3). Our results allow to both generalize physiological conclusions hitherto restricted to guard cells of *AtBAG4*-expressing plants [29] as well as to align cellular findings with effects previously observed with *BAG4* overexpression in different plants.

## Discussion

### Single cell tracking analysis pipeline allows extraction of crucial cell expansion parameters

Quantitative microscopy on single cells is frequently applied on animal cells e.g. for drug discovery screenings while this technique is not applied on plant cells to a similar extent. We have approached to expand the application of this technology using protoplasts, plant single cells which can be released from almost all plant tissues by enzymatic lysis of the cell wall. Depending on suitable media and plant growth regulator composition, cultivated protoplasts have the potential to proliferate, generating microcalli and finally whole plants. We were able to quantify various developmental properties of thousands of protoplasts by cultivation in multi-well-plates and the application of high-throughput automated microscopy coupled with image processing pipelines. Here, we focused mainly on early protoplast responses, cell expansion prior to the initiation of proliferation as we intended to study cell growth properties without the effects of shape-compromising cell walls. We used tobacco leaves as a widely used system for protoplast isolation and compared the properties of wild-type protoplasts with those expressing the antiapoptotic protein *AtBAG4* which is reported to alter various physiological parameters in different plant systems.

Our analysis pipeline was able to distinguish different protoplast subpopulations, corresponding to responding and non-responding cells which emerge after cultivation, and to extract their growth features such as expansion rate and response rate. This revealed that *AtBAG4* expression increased the fraction of protoplasts which responded with positive area change compared with wild-type protoplasts. Moreover, *AtBAG4*-expressing protoplasts showed an increased cell expansion rate compared with wild-type protoplasts, as well as a higher relative proliferation rate after continued cultivation. Interestingly, our results on single-cell responses can be associated with physiological data obtained with *BAG4-*expressing plants as discussed below.

### Automation of single-cell analysis workflow

Cell population analysis derived from single-cell tracking is very powerful in identifying phenotypes of interest [3]. Most methods in physiological and anatomical studies of cellular specialization rely on fluorescent protein markers and involve tracking and isolation of cell populations of particular identity [9]. However, such approaches clearly possess limitations and provide a biased analysis. Typical lifetime of a marker is much shorter than the developmental timeline of a cell, making it, as a result, difficult to track single cells over longer period of times [16]. In addition, protoplast development is not only dynamic but also progresses continuously, from isolation to differentiation, through multiple developmental stages. Therefore, markers labeling cellular components at the initial stage might lose its efficacy and introduce noise as the cell navigates through the complex landscape of development. Marker dilution to cell expansion also possess a problem as good markers for crucial cell populations are missing in some cases [9]. In contrast, quantification of cell morphology, which includes cell size and shape, monitored by bright field/gray scale images obtained from microscopic image acquisitions offer a powerful approach to marker-free single cell profiling. Due to the marker-free property, experiments are far less laborious experimentally and are applicable throughout various taxa, moreover such approaches also provide an unbiased view of cellular organization.

Recently, many tools have been developed which can identify and segment the boundary of single cells in grayscale microscopy images [16]. While some of these tools confound multiple image analysis techniques, others are based on deep/machine learning algorithms. There are also tools that can track single cells over time and perform analysis. Most of these tools are available as separate software that perform only one action, i.e., either segmentation, tracking or analysis [16]. Here we present a pipeline that combines on one platform cell segmentation, tracking, and analysis. Single cell segmentation in this pipeline is performed directly in grayscale microscopy images using U-Net, which is a powerful deep-learning solution for quantification tasks such as cell detection and shape measurements in biomedical image data [10]. In experiments extending over many days, due to repeated movement of the plate, cell positions are likely to be disturbed. The cell tracking module in our pipeline is able to track cells with slight shifts in cell position across time. Finally, the pipeline allows cell population analysis derived from statistics based on single-cell tracking. Such per-cell measurement based population analysis approaches quantify cell changes over time while maintaining information on heterogeneity in cell population [34].

### Universal function of BAG4 in controlling cell expansion

We have applied our pipeline on protoplasts expressing *AtBAG4* which was identified as an interaction partner of KAT1 assuming altered cellular properties [29]. In *AtBAG4-*expressing lines and mutants, altered regulation of cellular potassium levels was concluded from changed stomatal guard cell opening properties. This was molecularly explained by a BAG4-mediated procession of KAT1 during its way through the secretory pathway prior to its insertion into the plasma membrane. As the same family of K^+^ channels regulates K^+^ conductance also in most other cell types and protoplast expansion mainly depends on auxin-mediated regulation of ion channels causing enhanced abundance of intracellular K^+^ and Cl^−^ ions followed by passive water uptake, it is conceivable to expect a similar function of BAG4 on KAT1 and thus expect altered intracellular potassium concentrations in other cells as well [7]. However, approached to investigate this potential effects in cells embedded within tissues is difficult as altered cellular growth properties affected by accelerated K^+^ flux might readily be compensated by superior architectural requirements of leaves and finally might not affect cell size in plants with altered BAG4 levels.

However, as we show, leaf cells deprived from their volume-restricting cell walls, show altered properties when *AtBAG4* is expressed which aligns with similar properties determined for guard cells concluded from physiological experiments [29]. Increased abundance of KAT1 channel proteins similar to *AtBAG4*-expressing guard cells would result in accelerated K^+^ uptake, followed by faster volume changes through passive water influx just like we observed in *AtBAG4*-expressing protoplasts when compared with the wild type. Accelerated cellular K^+^ influx might also help to explain the enhanced resistance towards salt and drought stress which is observed in *AtBAG4-*expressing plants [21] although there are alternative explanations which relate to the function of BAG4 as an anti-apoptotic protein (see below).

### Different single-cell properties relate with phenotypic alterations in plants

While the link between BAG4 and ion homeostasis in guard cells—and as we impose in this work in other leaf mesophyll cells as well—is a recent finding, members of the plant BAG protein family are functionally associated with stress resilience and programmed cell death (PCD) in plants. PCD represents an evolutionary conserved pathway occurring in animals and plants which is also involved in many physiological processes in plants such as the controlled elimination of cells during growth and development and the hypersensitive reaction following abiotic and biotic stresses [30]. In mammals, the family of BAG proteins was initially identified as interaction partners of the antiapoptotic protein Bcl-2 and function similarly in preventing apoptosis when present in higher abundance [54, 57]. While default approaches to identify potential BAG homologues in plants using primary protein sequence identity as criteria were unsuccessful, seven structurally similar proteins were identified in *Arabidopsis* which share a common C-terminal BAG domain [8, 11]. Among these proteins, the function of BAG4 is most intensively investigated by overexpression and knockout approaches although biochemical mechanisms are largely unknown.

PCD is also induced in consequence of plant cell exposure to *Agrobacterium tumefaciens* during genetic engineering and reduces the transformation efficiency significantly. Interestingly, PCD was reduced and in consequence the transformation rate was strongly increased if the mammalian antiapoptotic genes *Bcl-2* were coexpressed in banana cell suspension cultures; moreover transient transgene expression rates were similarly improved when *AtBAG4* was coexpressed in tobacco [25, 40]. Moreover, cold stress treatment induced apoptosis-like cell death in wild-type tobacco plants while this was not observed in *AtBAG4-*expressing tobacco plants [20]. These results corroborate the involvement of the antiapoptotic genes including *AtBAG4* in promoting cell viability which is especially required after biotic and abiotic stress situations.

### Biotechnological applications of plant single-cell analysis tools

The enzymatic maceration required to release protoplasts from plant tissue generates various reactive oxygen species which are thought to exert oxidative stress to protoplasts [41]. This is maybe a major cause for the high rates of cell deaths which is usually observed upon protoplast isolation, despite the development of a variety of different complex cultivation media and optimization of plant growth regulators [18, 24]. As we show in this work, isolated protoplasts expressing *AtBAG4* exhibit a higher fraction of viable cells, identified as cells responding with increased cell area when compared to wild-type protoplasts. It is reasonable to assume that this is due to AtBAG4-mediated improvement of stress resistance thus allowing to associate single-cell properties with those observed in plants. For instance, tobacco lines expressing *AtBAG4* were shown to exhibit increased tolerance to UV light stress and the oxidants menadione or paraquat [20]. It thus seems conceivable to exploit single-cell systems for the identification of genes which confer improved stress resilience, e.g. by high-throughput screening of protoplasts from different genotypes exposed to a variety of different stresses mimicking biotic stresses on a whole plant level (e.g. salt, drought). Such approaches could accelerate development of new traits with improved agronomical properties as they bypass sophisticated and costly plant studies.

Although stress imposed on protoplasts during isolation results in severe cell losses, the cellular stress response which is induced via the lysis process is considered as an essential trigger for a stochastic expression of genes. This includes also key genes which finally enable protoplasts to reprogram and initiate proliferation [58]. As we show, *AtBAG4* expression in tobacco increases the fraction of cells responding with positive expansion which indicates their viability, thus presence of *AtBAG4* apparently increases the capacity to cope with stresses associated with the isolation and cultivation procedure. Confirmatory, we also observed a higher proliferation rate of *AtBAG4-*expressing protoplasts. Similar to the stress-protective effect of BAG4 observed with plant tissues as discussed above, this could be exploited for equivalent single-cell applications for instance via *AtBAG4* coexpression during single-cell gene editing using CRISPR/Cas9 in order to increase regeneration efficiency in protoplast in vitro approaches or support regeneration rate in equivalent approaches performed with protoplasts from recalcitrant taxa which usually fail to proliferate at all.

## Methods

### Generation of transgenic tobacco lines

For the backbone vector *pCAMBIA2300-35S*, the *CaMV-35S* promoter was amplified from the vector *mAV* and cloned into the vector *pCAMBIA2300-bar*. *pC2300-35S-AtBag4* was constructed by PCR amplification of the coding sequence of *AtBAG4* (accession no. *NM_115037.7*), with the *nos* terminator sequence, as present in the vector *pCAMBIA1305-Gt1-AtBAG4-nosT*, and inserted downstream of the *CaMV-35S* promoter in the vector *pCAMBIA2300-35S* by Gibson isothermal assembly [13]. For *pC2300-35S-eYFP-AtBag4*, *eYFP* was amplified from the plasmid *pGEN047-eYFP*, fused with the PCR fragment *AtBAG4-nosT* and cloned into *pCAMBIA2300-35S* by Gibson isothermal assembly. All constructs were verified by sequencing. Plasmids were transformed into *Agrobacterium tumefaciens* (strain LBA4404) and leaf explants from tobacco (*Nicotiana tabacum* L. cv. “Petit Havana” SR1; [32] were transformed as described [36]. Phosphinotricin was used with 2 mg/l for selection of transformants and with 1.5 mg/l for rooting.

Seeds from homozygous plants were sawn on MS agar and cultivated under long day conditions (16 h light–8 h dark) at 22 °C with 100 µmol photons m^−2^ s^−1^. Total RNA isolated from leaves was used to determine *AtBAG4* expression levels according to Maass et al. [31]. Specific *AtBAG4* mRNA levels were quantified by real-time RT-PCR using the primer pair *AtBAG-F* (GGA CCC GGG ATG ATG CAT AAT TCA AC) and *AtBAG-R* (CTC TAG TCG ACT CAG TCA AAT TTC TC) and SYBR green with *18S* rRNA levels for normalization. For *18S* rRNA quantification, the eukaryotic *18S* rRNA endogenous control kit (Thermo Fisher) was used. The relative quantity of the transcripts was calculated by using the comparative threshold cycle method [28]. Data were normalized first to the corresponding *18S* rRNA levels and then expressed as relative to the wild-type transcript levels.

### Protoplast isolation

Seeds from homozygous plants were sawn on MS agar and subcultured three subsequent times prior to first protoplast isolation as described in Lin et al. [27]. Protoplast isolation was performed from 2–3 young leaves of selected homozygous lines essentially using enzymatic digestion of leaf cell walls followed by filtration and density gradient centrifugation as described in Schnorf et al. [47]. The final protoplast pellet was resuspended in Kao medium [24] containing 0.4 M glucose, the auxins 1-naphthaleneacetic acid (NAA; 1 mg/l) and 2,4-D (0.2 mg/l) and the cytokinin 6-benzylaminopurine (0.5 mg/l). This medium is referred to as K8.

### Protoplast immobilization and microscopy

Protoplast density was determined by cell counting in a Fuchs-Rosenthal chamber. For immobilization, protoplasts were mixed with equal volumes of 1.2% (w/v) low melting agarose in K8, prewarmed at 40 °C and immediately pipetted into a 96-well plate (Ibidi GmbH, Munich, Germany), prewarmed at 34 °C with 125 µl per well. The plate was immediately centrifuged (2 min, 20 g, 25 °C) and incubated at 4 °C for 5 min. 200 µl of cultivation medium was added on top of each well and the protoplasts were cultivated at 22 °C in the dark.

An automated microscope (more, Till I.D. GmbH, Munich, Germany) was used for the analysis of protoplast development and for the detection of YFP fluorescence. Transillumination was recorded with 10 × 0.45 objective (Zeiss); epifluorescence was recorded after excitation with single-mode diode lasers (iBeam smart, Toptica) with excitation of 510 nm (YFP)/green emission filter. Image acquisition was performed using the SIAM software (Till I.D. GmbH, Munich, Germany).

### Image processing and data analysis

A detailed description on the software version and requirements and on individual steps on how to execute the analysis pipeline is provided in Additional file 1. All the scripts and codes used in the analysis pipeline, additional downloaded plugins used in processing the images as well as sample data can be found in our Github page https://github.com/jodawson/cell_seg_tracking_analysis.

Image processing was performed with Fiji [46] an image processing package distributed by ImageJ2 [44]. Raw tile image stacks were reconstituted to full-well images using the stitching plugin [42] and minor shifts within different time point recordings were corrected using the template matching and slice alignment plugin [56]. Image segmentation was performed with U-net [10]. For estimation of the protoplast proliferation, segmented objects were considered as proliferating microcalli if the object size after segmentation was larger than 4000 px, determined at DAI9 (corresponding to 40 µm diameter). Object numbers were normalized to cell number determined at DAI1 after subtracting background defined as objects with areas larger than 4000 px at DAI1, revealing the relative proliferation rate.

### Tracking script development

The automated image processing and subsequent statistical data analysis pipeline consists of five blocks, the (i) image pre-processing block, (ii) image segmentation block, (iii) image post-processing block, (iv) cell tracking block, and (v) the statistical data analysis block. Using this custom developed pipeline, fully automatized growth analysis of single cells in multiple images of different wells between any two time points was performed.

(I) Image pre-processing block
  The image analysis is built exclusively in java scripting language and is integrated into ImageJ [44, 46]. Running the image analysis part of the pipeline results in the automatic execution of a series of interlinked steps. The experimental data is stored such that images (in.tif format) were sorted according to different wells and time points. For example, corresponding to well 1B, there are two images which were named as ‘1B_TP1.tif’ and ‘1B_TP2.tif’, where TP1 and TP2 correspond to time point 1 (DAI0) and time point 2 (DAI1), respectively. The following steps were implemented in this block:
  - The cell positions were aligned among all chronological images in order to correct for slight positional displacements of protoplasts using the template matching and slice alignment plugin [56]. The pre-processed image files were saved in the original folders/directories for each well and corresponding time point.
  - After correcting for the shift in the images recorded between two time points each image was cropped into nine sections of equal dimensions, i.e. 3 × 3 to enhance processing speed and restrict memory usage.
  - The contrast of each section of the cropped image was increased using the in-built ImageJ plugin ‘Normalized Local Contrast’. For execution of this plugin, we used the following values: block_radius_x = 90, block_radius_y = 90, standard_deviations = 1, center. This process not only increases the contrast of the cell objects in the image but it also smoothens the shading of light across the border of tiles within each section.
  - The contrast of the cropped section of the image is further enhanced using the in-built ImageJ plugin ‘Enhance Contrast’. We used the following parameter values: saturated = 0.8, normalize.
  - Since each image at first place resulted from stitching multiple tiles together, there is a clear mark of tile border both in vertical and horizontal direction. To remove these vertical and horizontal lines, resulting from the borders of the tiles, each cropped section of the image was further processed using the in-built ImageJ plugin ‘Bandpass Filter’. The following parameter values were used for executing this plugin: filter_large = 100 filter_small = 3 suppress = Horizontal tolerance = 5.
(II) Image segmentation block
  After each section of the cropped image is pre-processed as described in the ‘Image pre-processing block’, it passed on to U-net. U-net is an open source, deep learning based software for biomedical image segmentation [10]. Processing via U-net results in a segmented binary image corresponding to the cropped section of the experimental gray scale pre-processed image. The binary image is an image that consists of pixels that can have exactly one of the two colors black or white. Generally white corresponds to the region occupied by each individual cell, and black corresponds to the image background. U-net identifies with remarkable accuracy each individual cell in the image. This process of identifying cells and differentiating them from the background is known as segmentation. The resulting binary image is processed, so as to fill any holes using the in-built ImageJ plugin ‘Fill Holes’: A new folder with the same name as the image Is created, for example ‘1B_TP1’. The resulting binary image of each cropped section of the image is then saved in this folder. As a result, it is expected at the end to have 9 binary images, each corresponding to the cropped section.
(III) Image post-processing block
  The binary image of each cropped section of the original image is stitched using the in-built ImageJ plugin ‘Grid/Collection Stitching’. This process results in a segmented binary image which has the same dimension as the original raw experimental image. This binary image is then processed via ‘Watershed’ function. Watershed splits multiple cells that were identified as one cell object in the U-net based segmentation step. This usually happens when the cells are too close and touch each other forming clumps or multicellular aggregates. In order to remove such cell aggregates from the cell tracking analysis we adopted the speckle inspector plugin in ImageJ to identify cells which formed clusters, by defining cells in such a cluster as speckles, and by comparing the original segmented image with the corresponding watershed image [4]. Finally, the information of each and every segmented cell in the binary image, such as their position coordinates, area and circularity, is obtained by using ‘Analyze Particles’ function. Running ‘Analyze Particles’ produces a result file (.txt format) which contains a list of all the individual segmented cells and their corresponding position (*x* and *y*) coordinates, area and circularity.
(IV) Cell tracking block
  After the image pre-processing, segmentation and post-processing is completed, the next step involves tracking of single cell between two time points. A custom-built code developed in Matlab performs this task. Currently, our cell tracking is limited to tracking single cells between two time points. This will be extended, in the future, to track cells over multiple time points. Cell tracking of a cell in the first time point involves successfully identifying this cell in the second time point. We implement cell tracking based on the Euclidean distance between the cell centroid coordinate at the first time point and the second time point. The Euclidean distance is given by ${d}_{ij}=\sqrt{({x}_{i}^{\mathrm{TP}1}-{x}_{j}^{\mathrm{TP}2}{)}^{2}+({y}_{i}^{\mathrm{TP}1}-{y}_{j}^{\mathrm{TP}2}{)}^{2}}$, where $i$ denotes the index of a cell in the first time point TP1 and $j$ denotes the index of a cell in the second time point TP2. A cell in the first time point is successfully identified in the second time point if the Euclidean distance is smaller than a threshold. The execution of this step of the pipeline generates a result file. The result file lists an array of all the cells of the first time point, and their corresponding features (such coordinates, area etc.), followed by an array of all the cells that were tracked from the first time point, and their corresponding features.
(V) Statistical data analysis block
  From the result file containing the information of cells tracked between two time points various different statistical analyses, such as those reported in this work, were performed in Matlab using custom built codes.

## Supplementary Information

**Additional file 1.** Workstation specification, software tools and flow of code execution.

**Additional file 2.** Additional figures.

## Data Availability

The complete datasets used and/or analyzed during the current study are available from the corresponding author on reasonable request.
