# Supplementary material for: Determination of protoplast growth properties using quantitative single-cell tracking analysis
Source: Plant Methods. 2022 May 18;18:64. doi: 10.1186/s13007-022-00895-x (PMC9118701; doi:10.1186/s13007-022-00895-x)
Supplement: Supplementary file 1 — Additional file 1. Workstation specification, software tools and flow of code execution. [file 13007_2022_895_MOESM1_ESM.pdf]

Additional File 1
Workstation specifications and Software tools used

- **Workstation**: We performed all our analysis on a Linux workstation with following specs:
  - CPU: Intel i7 quad core 7th gen
  - RAM: 32GB
  - GPU: Nvidia GTX 1080Ti
  - GPU supported Unet installation.
- **Unet**
  - All the instructions and the necessary steps for installing Unet, including video tutorials, can be found here: https://lmb.informatik.uni-freiburg.de/resources/opensource/unet/
  - The Unet Github page: https://github.com/lmb-freiburg/Unet-Segmentation
- **ImageJ**
  - Version ImageJ 1.52P (linux version 64 bit) was used
  - ImageJ can be downloaded from: https://imagej.net/software/fiji/downloads
  - All the extra/additional plugins used for operations such as: Template matching, EDM Binary operation, Speckle Inspector, are uploaded on our Github page at this location: https://github.com/jodawson/cell_seg_tracking_analysis/tree/main/ImageJ_plugins
  - These plugins should be copied into the user's ImageJ plugin folder.
  - The latest version of these plugins can also be downloaded from developers own website (in this case compatibility with our own ImageJ version not guaranteed):
  - Template Matching and Slice Alignment plugin: https://sites.google.com/site/qingzongtseng/template-matching-ij-plugin
  - Biovoxxel plugin (for EDM binary op's and Speckle Inspector): https://imagej.net/plugins/biovoxxel-toolbox
- **Matlab**
  - Version 2018a used
  - All the necessary functions used to plot various figures are present in our Github folder located at: https://github.com/jodawson/cell_seg_tracking_analysis/tree/main/Plot_functions
  - Add the path of the folder containing these functions to your Matlab path
  - All the various codes used for analyzing the data are uploaded on our Github and supported by a extensive Readme instruction file at this location: https://github.com/jodawson/cell_seg_tracking_analysis

Flow of code execution: General Remarks

- **Nomenclature of image files:**
    - All image files obtained from microscopy should be in the '.tif' format
    - Each image file should be named in the following manner: Experimentcode_WellrowWellcolumn_Timepoint
    - For example:
        - Our images were recorded in a 96-plate well (rows=B, C, D and columns=1, 2, 3..., 12);
        - The experiment code for one particular experiment was given to be: Tob19C
        - Each timepoint was labelled as TP, i.e., DAI0 labelled as TP1, DAI1 as TP2, DAI2 as TP3, DAI3 as TP4
        - Therefore, an image with filename 'Tob19C_1B_TP1' means recording of the well 1B on DAI0 of the experiment Tob19C
    - Any deviation from this nomenclature for image filenames will require adjustment in the provided source codes
- **Data architecture required for running the scripts:**
    - All the image files of all the wells and all the timepoints from one experiment are stored in one parent folder (for clear identification, name the parent folder with the experiment code mentioned in the preceding section). For example, the folder hierarchy for the experiment labeled experiment code Tob19C will be Tob19C>{Tob19C_1B_TP1.tif, Tob19C_1B_TP2.tif,...,Tob19C_2B_TP1.tif, Tob19C_2B_TP2.tif,..., Tob19C_12D_TP2.tif}. Deviations from this hierarchy will require some small adjustments to the tracking code so that the correct image files are found.
    - The parent folder that contains all the image files also contains a text file in .txt format (such as the uploaded file **well_label_b.txt** in our github page) which contains a list of all the well names (such as 1B,2B...) with one well name per line.
    - A sample data folder containing the image files and the well info file is uploaded on our Github page at:
      https://github.com/jodawson/cell_seg_tracking_analysis/tree/main/Test_Data/Tob19C
      (Since our image files are greater than 25Mb (1 Gb) we uploaded a cropped smaller version of the images)

Flow of code execution: Steps

(0) For each image: Within each well across timepoints (DAI0, DAI1, DAI2...) images should be aligned to correct for any major shifts that can occur during recording. This alignment is done in ImageJ using the plugin 'Template Matching'. Detailed information about how to perform the template matching and the necessary plugins required to run this on ImageJ are provided here: https://sites.google.com/site/qingzongtseng/template-matching-ij-plugin. The resulting aligned images **should replace** the original images in the parent folder.

(1) Start ImageJ and open and run the IJ code 'Process_DIC_Tobacco_3.ijm' (https://github.com/jodawson/cell_seg_tracking_analysis/blob/main/Process_DIC_Tobacco_3.ijm)

- When you start the code execution a GUI will appear that will ask for the directory/folder where all the image files (in .tif format) are stored, select the correct folder.
- The above process initiated by running '**Process_DIC_Tobacco_3.ijm**' creates within the parent folder multiple sub-folders, one sub-folder for each image (a sub-folders name is the same as its corresponding image) in which the deep learning based image segmentation results are stored.
- Each sub-folder of its corresponding image, i.e., the image of a particular well and timepoint, consists of: (a) ROI file of the segmented cells, (b) Binary image (.tif format) of segmented cells, (c) Result file (.txt file) containing various measurements of the segmented cells in the binary image.

(2) In the next step, load another ImageJ code '**Process_DIC_CellCluster_Filter.ijm**' (https://github.com/jodawson/cell_seg_tracking_analysis/blob/main/Process_DIC_CellCluster_Filter_.ijm).
This code runs through each sub-folder (that corresponds to each image file) and further processes, using ImageJ plugin 'Speckle Inspector', the binary image stored in that sub-folder and saves a new binary image in which all clusters of cells touching each other are removed. The new binary image is analyzed and the new result and ROI files are stored in the .txt and .zip format, respectively.
(3) Third step involves running the Matlab code '**cell_tracking_analysis.m**' (https://github.com/jodawson/cell_seg_tracking_analysis/blob/main/cell_tracking_analysis.m).

- Running this code will prompt the user to enter the first time point, whether 1 or 2 or 3 etc. (1 corresponds to TP1, 2 to TP2 etc.) and the folder, this would be the parent folder, in which the image files and their corresponding sub-folders are stored.
- After successful execution of this code, the parent folder will be populated with multiple text (.txt) files for each well. For example, for the data we uploaded of the well 10G for TP1 and TP2 we have result following files:
- 10G_TP1_TP2_N_cell.txt: This contains in the first column total number of cells that were tracked between TP1 and TP2, and in the second column the total number of tracked cells with area growth greater than 2.

- 10G_area_circ_TP1_TP2.txt: contains in column 1 area of cells at TP2, column 2 area of cells at TP1, column 3 circularity of cells at TP2, column 2 circularity of cells at TP1.
- 10G_area_circ_gt2_TP1_TP2.txt: contains the same information in four columns but only of cells that have are growth rate of greater than 2.

(4) Fourth step involves running the Matlab code '**statistical_data_analysis.m**' (https://github.com/jodawson/cell_seg_tracking_analysis/blob/main/statistical_data_analysis.m).

After this code is successfully executed, the Matlab workspace will be populated with various tables, arrays and cell structures which are used to plot various distributions, statistical measurements etc. which are reported in this article.

- As an aid to understand how the data in the Matlab's workspace generated after the execution of step (4) is used, a Matlab script file '**manuscript_figure_plotting_script.m**' (https://github.com/jodawson/cell_seg_tracking_analysis/blob/main/manuscript_figure_plotting_scripts_Tob19C_gthb.m) is uploaded. In this file various Matlab scripts used to generate each figure reported in this article is presented.
