# Supplementary material for: Determination of protoplast growth properties using quantitative single-cell tracking analysis
Source: Plant Methods. 2022 May 18;18:64. doi: 10.1186/s13007-022-00895-x (PMC9118701; doi:10.1186/s13007-022-00895-x)
Supplement: Supplementary file 2 — Additional file 2. Additional figures. [file 13007_2022_895_MOESM2_ESM.pdf]

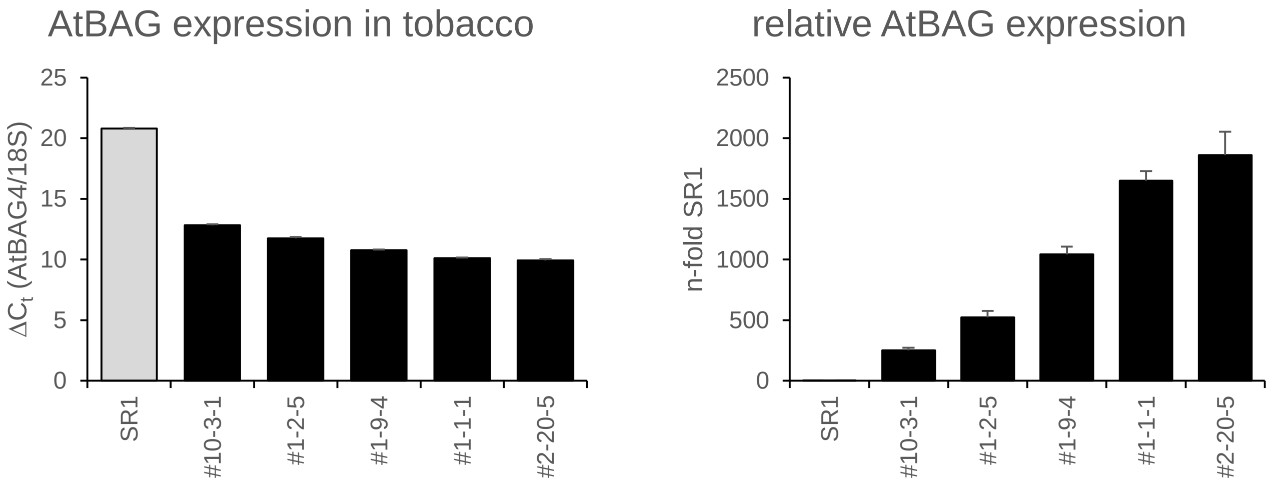

Additional Figure 1: *AtBAG4* expression levels in transgenic tobacco lines
Nicotiana tobacum, variety SR1, was transformed with *AtBAG4* (lines #1-2-5, #1-9-4, #1-1-1) and *YFP-AtBAG4* (lines #10-3-1 and #2-20-5) under control of the *CaMV-35S* promoter, respectively, and *AtBAG4* transcript levels were determined by RT-qPCR in leaves of 3 week old plants. ΔCt values of *AtBAG4* versus *18S* rRNA used as internal control (A) indicate low background amplification of endogenous tobacco *BAG* by the *AtBAG4*-specific SYBR primer pair. Normalized expression levels were expressed relative to SR1. Lines #1-1-1 and #2-20-5 were used for further analysis. Results are means ± SD from three technical replicates.

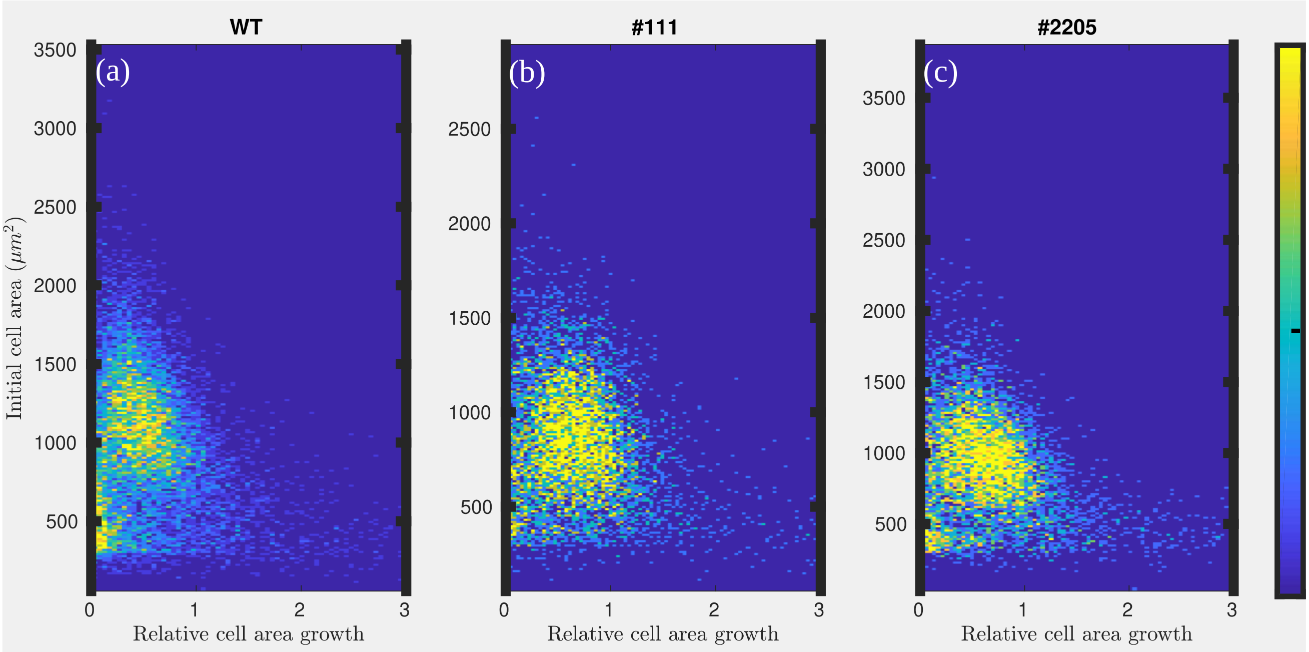

Additional Figure 2: Density scatter plot of initial cell area versus the relative cell area growth
The density scatter plot of initial cell area versus the relative cell area growth of the cells tracked between DAI0 and DAI2 for (a) wild type, (b) #111 and (c) #2205 *AtBAG4* expressing lines. Relative cell area growth of 0 implies that the cells have not increased their area in the specified time period, i.e., between DAI0 and DAI2, whereas relative cell area growth of 1 implies that the cells have doubled their area.

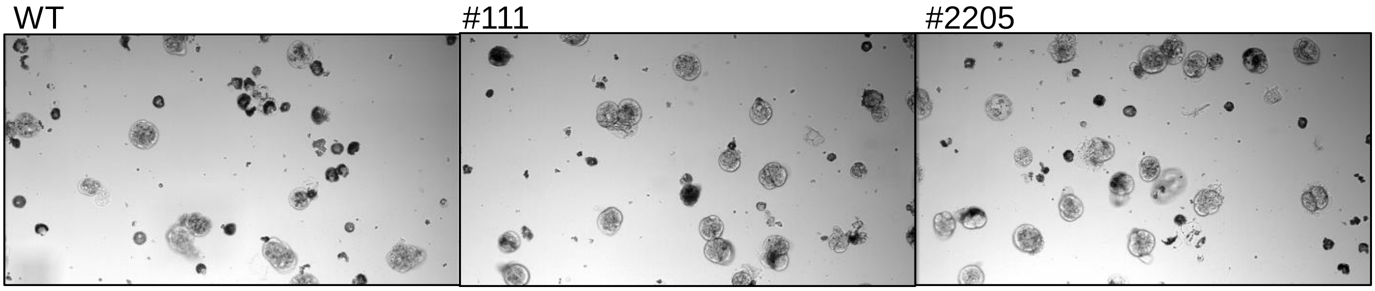

Additional Figure 3: Bright field images of proliferating tobacco protoplasts
Tobacco protoplasts were isolated from Wt and two *AtBAG4* overexpressing lines, immobilized and overlaid with proliferation medium for 5 days. Shown are representative bright field recordings.
